# A Guide to Cannabis Virology: From the Virome Investigation to the Development of Viral Biotechnological Tools

**DOI:** 10.3390/v15071532

**Published:** 2023-07-12

**Authors:** Niccolò Miotti, Alessandro Passera, Claudio Ratti, Mattia Dall’Ara, Paola Casati

**Affiliations:** 1 Department of Agricultural and Food Sciences—Production, Landscape, Agroenergy, University of Milan, Via Celoria 2, 20133 Milan, Italy; alessandro.passera@unimi.it (A.P.); paola.casati@unimi.it (P.C.); 2 Department of Agricultural and Food Sciences (DISTAL), University of Bologna, Viale Giuseppe Fanin 40, 40127 Bologna, Italy; claudio.ratti@unibo.it

**Keywords:** hemp, *Cannabis sativa*, virus, plant protection, viral vector

## Abstract

*Cannabis sativa* cultivation is experiencing a period of renewed interest due to the new opportunities for its use in different sectors including food, techno-industrial, construction, pharmaceutical and medical, cosmetics, and textiles. Moreover, its properties as a carbon sequestrator and soil improver make it suitable for sustainable agriculture and climate change mitigation strategies. The increase in cannabis cultivation is generating conditions for the spread of new pathogens. While cannabis fungal and bacterial diseases are better known and characterized, viral infections have historically been less investigated. Many viral infection reports on cannabis have recently been released, highlighting the increasing threat and spread of known and unknown viruses. However, the available information on these pathogens is still incomplete and fragmentary, and it is therefore useful to organize it into a single structured document to provide guidance to growers, breeders, and academic researchers. This review aims to present the historical excursus of cannabis virology, from the pioneering descriptions of virus-like symptoms in the 1940s/50s to the most recent high-throughput sequencing reports. Each of these viruses detected in cannabis will be categorized with an increasing degree of threat according to its potential risk to the crop. Lastly, the development of viral vectors for functional genetics studies will be described, revealing how cannabis virology is evolving not only for the characterization of its virome but also for the development of biotechnological tools for the genetic improvement of this crop.

## 1. History of *Cannabis sativa* Virology

**Figure 1:**
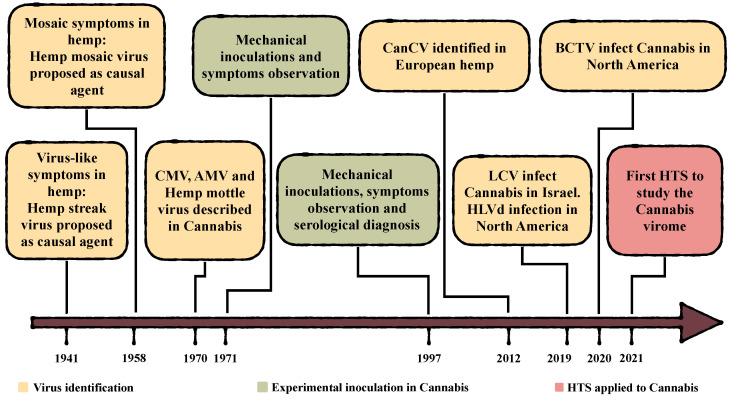
Chronology of major reports of virus-like cannabis diseases and viruses/viroid infecting *C. sativa*.

The earliest observations of *Cannabis sativa* viral disease are reported in the 40s and 50s of the XX century in Europe [1,2] (Figure 1) when fiber and/or seed crops were widely cultivated. These first reports indicate two symptomatologic conditions that were correlated to supposed viral infections, never formally demonstrated: the hemp streak virus (HSV) and the hemp mosaic virus (HMV). However, these reports were purely descriptive of symptomatology since no pathogenicity test was ever performed, and the viral etiology was never confirmed. A comprehensive analysis of these conditions has never been conducted, probably because hemp cultivation faced a period of regression due to the development of synthetic fibers and the use of other plants as a source for vegetable oil production in later years. In 1970 Schmidt and Karl [3] identified three viruses able to infect *C. sativa* plants and cause symptoms: the cucumber mosaic virus (CMV), alfalfa mosaic virus (AMV), and hemp mottle virus. While CMV and AMV were confirmed as infectious and pathogenic in later studies, the latter was never reported again. In the 1970s, Hartowicz et al. (1971) [4] performed viral mechanical transmission in *C. sativa* using isolates of twenty-two different viruses, and half were proven to infect inoculated plants. While this work was presented at the “North Central Weed Control Conference”, to our knowledge, a comprehensive and peer-reviewed paper reporting the methodology alongside the results obtained has never been published. A later publication [5] mentions this work and lists those viruses that resulted in symptomatologies in inoculated plants: tobacco ringspot virus (TRSV), tomato ringspot virus (TomRSV), tobacco streak virus (TSV), and CMV have been associated with mosaic symptoms and dwarfism, while AMV and the so-called “eunoymous ringspot virus (ERSV)” were only associated with mosaic. Of these, only TSV, CMV, and AMV were reported again in later works on viruses that infect *C. sativa*, while the others were no longer mentioned.

No new manuscripts on the topic were then published until 1997, when Kegler and Sparr [6] performed inoculations of different viruses in several European industrial varieties for seeds and fiber production (hemp) in a controlled environment. After six weeks from the first inoculations, systemic leaves of infected hemp plants were used for re-inoculations on test plants (*Nicotiana* spp. and *Chenopodium* spp.) using serological tests to verify viral infections. In this way, it was possible to identify the viruses described in Table 1, including CMV and AMV, and describe a range of symptom severity in hemp (Table 2).

More than ten years passed until a new virus infecting hemp was accidentally identified in 2012 [9]. The new Partitiviridae identified was proven to be widely endemic since it was detected in almost all varieties tested, but no correlation with symptoms was observed, so it was reported as cannabis cryptic virus (CanCV) (Table 1). In a follow-up study, characterizing the virus in other hemp varieties, Righetti et al. (2018) [10] confirmed that the virus is not associated with any symptoms (Table 2) and described a variable viral load depending on the plant analyzed. Vertical transmission has been demonstrated crossing both infected male plants and healthy females and healthy males and infected females. The resulting progenies were found to be 100% positive. Otherwise, mechanical transmission in *Chenopodium quinoa* and *Nicotiana benthamiana* test plants was proven to be ineffective as was the transmission via cross-grafting between rootstocks and scions from infected and not-infected *C. sativa* plants. In fact, members of the *Betapartitivirus* genus have no movement proteins so, in plants, their tropism occurs only with cell division [16].

The hop latent viroid (HLVd) has recently been increasingly associated with severe cannabis symptoms [14,15,17] (Table 2) that impair seed and fiber production in industrial varieties and cannabinoid contents in medical varieties. Infected medical cannabis plants can suffer vigor loss and a 50% up to 70% reduction in THC content. Furthermore, a survey carried out in California on 200,000 samples verified the presence of HLVd in 90% of cannabis-growing facilities. The potential estimated losses can therefore reach up to USD 4 billion loss per year in the United States of America alone [17]. Infected mother plants for agamic propagation act as a reservoir for the viroid that easily spreads to the clonal progeny. Moreover, HLVd diffusion is higher than field inspections would indicate because symptoms develop only in the late stages of infection.

Where the decriminalization and legalization of *C. sativa* for medical (Israel) and recreational (in some USA states) use have taken place, cultivated areas have increased, leading to favorable conditions for the spread of new viral infections. Lettuce chlorosis virus (LCV) was identified as a causal agent of leaves yellowing and stunted growth in Israel [11] (Table 2), symptoms that were previously only associated with nutrition deficiencies. In the USA, apical leaf deformations have been correlated with the presence of beet curly top virus (BCTV) [12,13] (Table 2).

Recently, Pitt et al. (2022) [8] confirmed that potato virus Y (PVY) infects *C. sativa* and demonstrated that it can be transmitted by aphids in a non-persistent manner. This makes PVY-infected cannabis fields potentially dangerous from a phytosanitary point of view because of the possible spread of the virus to other crops.

*C. sativa* virome investigation has recently found a new boost thanks to high-throughput sequencing (HTS) techniques. Chiginsky et al. (2021) [7] performed an Illumina NextSeq500 sequencing of several *C. sativa* plants showing virus-like symptoms. While aiming to characterize different BCTV strains and describe their spread among symptomatic plants in Colorado (USA), several viruses have been identified (Table 1), some even never reported in *C. sativa*. The genome of a new strain of TSV was partially reconstructed, and grapevine line pattern virus (GLPV), citrus yellow vein-associated virus (CYVaV), and opuntia umbra-like virus (OULV) were identified and reported for the first time infecting cannabis. Recently, Jarugula et al. (2023) [18] reported co-infections of BCTV along with HLVd and CYVaV after canonical reverse transcription–PCR analysis and HTS in Washington State (USA).

All accession numbers of viruses that have been detected through HTS in cannabis are listed in Table S1 in the Supplementary material.

In recent years, in addition to expanding knowledge of the cannabis virome, cannabis virology has focused on exploiting viral vectors to compensate for the lack of established tools for in vivo functional genomic analysis. With the expansion of genomic and transcriptomic resources, significant advances have been made in the understanding of cannabis genetics allowing the identification of regulatory genes responsible for valuable medical or agronomical phenotypic traits [19,20,21,22,23]. However, reverse genetics studies are required to validate bioinformatic predictions or to conduct molecular characterizations of protein effectors. The basic requirement of reverse genetics is to possess tools for targeted gene editing to vary or abolish the expression of a particular gene or to obtain specific mutations to its product. The use of sequence-targeted DNA endonucleases underlies genome-editing technologies such as CRISPR/Cas9, which are widely used in other crops of agricultural interest [24,25,26]. The repair of DNA double-strand breaks by non-homologous end joining or homology-directed repair (in the occurrence of a donor template) makes it possible to modify a single DNA base pair, larger genomic portions, or the regulation of gene expression [27]. For this to occur, the CRISPR/Cas9 complex must be delivered or expressed in callus-forming cells (e.g., by *Agrobacterium tumefaciens*-mediated transformation), which will then be regenerated in an adult plant. However, cannabis plants are described as recalcitrant to callus regeneration even though they are effectively transformed by *A. tumefaciens* [28]. Indeed, the production of transgenic plants is still not easy to achieve [15], and despite their significant potential, transgenesis and genome editing have been successfully employed in only a few works [29,30]. In this context, virus-induced gene silencing (VIGS) represents an alternative system to genome editing for functional genomics studies because it allows the down expression of target genes through post-transcriptional gene silencing (PTGS). Viral vectors engineered from cotton leaf crumple virus (CLCrV) [31] and tobacco rattle virus (TRV) [32] have been successfully used for the purpose of harboring portions of *C. sativa* gene sequences in their genome and promoting the silencing of visible markers such as phytoene desaturase (PDS) and magnesium chelatase subunit I (ChlI). These promising results encourage the use of VIGS for rapid reverse genetic screening by silencing specific genes involved in the regulation of specialized metabolites, disease resistance, plant development, and abiotic stress in cannabis.

## 2. Categorizations of Viruses and Viroid According to the Level of Threat

**Figure 2:**
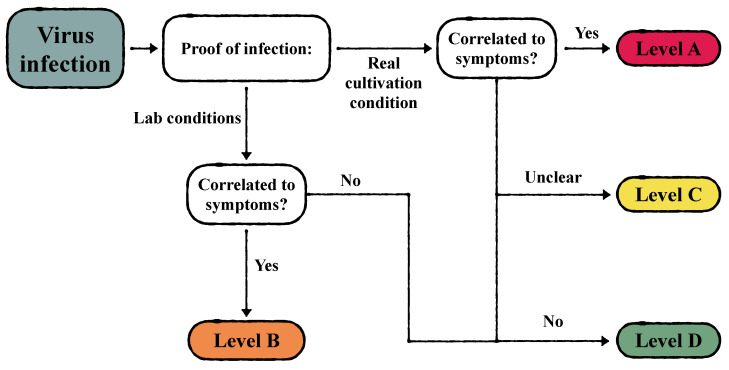
Graphic representation of the categorization of virus infection based on level of proof and correlation to symptoms.

Here, we propose a subdivision of viruses and viroids detected in *C. sativa* according to their threat level for cannabis cultivation, from the highest level A to the lowest level D (Table 2). The main pathological characteristics of each infectious agent were considered for classification into the four different threat levels: severity of symptom development and type of horizontal transmission mediated or not by vectors. Further criteria for classification among viruses is the natural occurrence of infection in cannabis by distinguishing infectious agents identified during field surveys from those inoculated artificially in lab environments (Figure 2). One should keep in mind that most of the available information on the biology of the viruses treated in this paper comes from studies conducted on hosts other than cannabis. Moreover, their role as causative agents of associated symptoms has never been formally demonstrated by means of infectious cDNA clones as suggested by Massart et al. (2017) [33] as an unequivocal method for defining the causal relationship between viral agents and diseases. Further studies would therefore be needed to characterize each member of the cannabis virome.

### 2.1. Level A

Level A of our classification includes viruses detected in the field/or greenhouse conditions, toward which, an epidemiological survey is active. These viruses can spread horizontally by vector or by agamic propagation and have been associated with significant yield losses in *C. sativa* as well as in other economically relevant crops.

BCTV, LCV, and HLVd belong to this threat level. Of note, these viruses and viroids have only been reported in recent years due to the favorable conditions for the spread of the pathogens that occurred after the intensification of *C. sativa* cultivation in the USA (BCTV and HLVd) and Israel (LCV). The obvious alterations in plant development and a significant reduction in plant yield made it necessary to investigate the etiological agent causing these diseases. In the early stages of vegetative growth, BCTV- and LCV-infected cannabis plants are characterized by leaf yellowing and chlorosis that can easily be interpreted as manifestations of abiotic stresses, such as improper fertilizer input [11,13]. This makes any field phytosanitary inspection difficult. Otherwise, in adult plants, BCTV appears to induce unequivocal and easy-to-identify viral symptoms such as mosaic, mottling, deformation, and wrinkling (Table 2), whose severity can reduce plant survival and subsequent crop production [13]. Analyses performed on symptomatic and asymptomatic plants in different Colorado counties have shown a strong correlation between the presence of BCTV and the curly top disease described, corroborating the causation hypothesis [18]. Growers’ concerns rely on the fact that the virus can widely spread in the fields and the symptoms caused by the infection can reduce flower mass and quality. Specifically, a survey conducted in twelve Colorado counties indicates an incidence of about 80% of infected plants with severe symptoms that can afflict more than 50% of their leaves [7].

With its eleven different strains [34,35], BCTV has a wide host range within dicot plants, infecting more than 300 species of 44 different plant families [36] and is considered a threat to different crop productions. It is transmitted by the beet leafhopper *Circulifer tenellus* (Hemiptera), in a persistent, circulative non-propagative manner [35]. Instead, LCV is transmitted by the whitefly *Bemisia tabaci* in a semi-persistent manner. Transmission trials verified that, once infected with the LCV, twenty asymptomatic plants developed different degrees of symptoms after infection. Pale green interveinal chlorosis of mature leaves after 20 days and yellowing streaks and dropping of foliage after 45 days were described [11].

HLVd infects dicots and was first described in hops, to which it causes altered cone development and the hyperaccumulation of alpha acids [37]. It was also isolated from Urtica dioica [38] and recently in *C. sativa* [14,15]. HLVd has no known vector: its horizontal transmission is mechanical (wounds or infected cutting tools) and it is unable to be transmitted through seeds or pollen [38]. HLVd in cannabis causes symptomatology colloquially referred to as “dudding”, which is characterized by brittle stems, reduced flower mass and trichomes, vigor loss and stunted growth, and a reduced rooting rate (Table 2) [14,15,39], which compromise the growth and productivity of infected plants. A comparison of the sequences of HLVd isolates from hops and cannabis revealed high conservation with only one mismatch between two isolates that still does not result in alterations in the RNA secondary structure [15]. Growers’ concerns about the viroid spread are because symptoms are not obvious and easily detectable, resulting in the difficult identification of infected plants and the impossible implementation of sanitation practices in the field. Some cannabis cultivars showed tolerance during the infection of HLVd, implying that symptom severity and expression could depend on the host genotype [17]. Prevention, based on sanitizing cutting tools, the screening of mother plants, and breeding for tolerant/resistant plants is the only effective way to reduce HLVd spread. A pre-print work highlighted the perfect homology between 19 nt of the HLVd and the 5′ coding region of COG7 [40], a gene involved in shoot apical meristem growth in Arabidopsis thaliana [41]. This under-reviewing work showed the downregulation of COG7 expression in infected cannabis plants, suggesting that RNA interference might be behind the HLVd pathogenicity. The ability of small RNAs derived from viruses and viroids to control genes involved in plant growth has already been reported [42]. Future studies on the cannabis RNA degradome may therefore elucidate the pathogenicity mechanisms of viruses described in this threat level.

### 2.2. Level B

Level B includes viruses of which no recent reports of natural infection in open-field or greenhouse plants are available, but whose ability to infect *C. sativa* and induce symptoms has been proven through experimental inoculations in protected environments (Table 2). Most of these viruses are known to possess a vector in other well-studied and -characterized crops, but these vectors have yet to be verified in *C. sativa* cultivation contexts. For this reason, these viruses are classified here as potential threats and are: AMV, ArMV, BBWV, CMV, PVX, PVY, RRSV, and TSWV. These findings come from the work of Kegler and Spaar [6], who performed mechanical inoculation tests in different industrial cannabis varieties from Europe. Among the varieties tested, USO 31 was found to be the most susceptible to viral infection and symptom development, although their descriptions give no indication of the impact on plant yield. The symptoms described, such as mosaic, wrinkling, and mottling (Table 2), can likely reduce the yield of cannabis plants in both industrial and medical productions. Most of these viruses have not been further characterized or reported a second time in cannabis plants. AMV and CMV deserve a separate mention because, although they have never been reported in more recent surveys and HTS screenings, they were found once in the 1970s [3], infecting field-grown cannabis plants. Also taking into account their potential ability to be transmitted through seeds (demonstrated in other crops [43,44]), CMV and AMV deserve special attention and consideration as real threats.

The same mention can be made of PVY, whose biology in *C. sativa* has been further characterized recently. Pitt et al. [8] extensively studied the interaction between PVY and the aphid *Phorodon cannabis* Passerini, describing viruliferous insect feeding behavior and their ability to transmit the virus in a non-persistent manner. Cohorts of twenty aphid transmission assays have proven efficiency in the transmission of PVY of 96% on host plants (*C. sativa*) and 91% on non-host plants (*Solanum tuberosum* L.), while the efficiency of single-aphid transmissions were lower, respectively, 63% and 19%. Viruliferous aphids, therefore, can mediate PVY spread, not only in cannabis fields but also in nearby susceptible crops, underling a possible real phytosanitary threat.

### 2.3. Level C

The members of this level are viruses detected in symptomatic cannabis plants from fields or greenhouses; however, none of these have been inferred as virulence determinants, so, to date, it is impossible to define them as pathogenic or asymptomatic. Some of these viruses have been characterized in other cultures but not in *C. sativa*. Indeed, all biological information defining an infection, such as pathogenicity association and modes of transmission, are lacking for this host. Given the current understanding, members of this category are therefore considered moderately dangerous to cannabis crops and should be actively monitored until evidence of pathogenicity is provided. Whenever that happens, a change in risk level categorization is recommended, classifying viruses in level A if they are pathogenic or in level D if they are not.

Viruses belonging to this level are: CYVaV, GLPV, OULV, and TSV. All were identified by Chiginsky et al. (2021) [7] who performed HTS screening on pools of five BCTV-positive symptomatic samples from different counties in Colorado, USA. Both TSV and GLPV belong to the *Bromoviridae* family, while CYVaV and OULV are currently unclassified and are here referred to as umbravirus-like associated RNAs (ulaRNAs). The identified TSV, interestingly, shares only 80–83% nucleotide identity with the nearest Genebank accession, making it a newly evolved genotype in *C. sativa*. In addition to BCTV, the viruses described in this work could be found in mixed infections with each other. This, unfortunately, cannot be determined since the HTS analysis was conducted on sample pools. The study and characterization of possible coinfections of viruses sharing the same epidemiological area could give information on the synergistic effects of symptom worsening. The presence of two ulaRNAs in cannabis plants raises the question of how their transmission occurred. They have a similar genome organization: CYVaV encodes only two proteins with replicative activity, while OULV possesses a third ORF (open reading frame) with a currently unknown function [45]. Despite the lack of silencing suppressors and movement proteins, ulaRNAs can replicate and spread systemically in the infected plant [46]. Like Umbraviruses, OULV and CYVaV do not form conventional virions in infected tissues and are unable to be transmitted on their own. While it is known that Luteoviridae provides the coat protein for trans-encapsidation of the Umbravirus RNA and allows its persistent transmission through an aphid vector [47,48], no helper virus has currently been associated with ulaRNAs. Indications that ulaRNAs must also possess a helper virus come from the finding of a Polerovirus-like virus along with OULV in aphids feeding on opuntia [49], even though evidence on actual transmissibility has never been provided. Thus, it can be hypothesized that, in cannabis as well as in other hosts, the occurrence of ulaRNAs dissociated from helper viruses comes from their loss after aphid transmission or during plant vegetative propagation. Follow-up studies remain necessary to elucidate the biology of ulaRNAs in cannabis, as the characterization of their transmission is required for the establishment of pest prevention practices.

### 2.4. Level D

Viruses detected both in symptomatic and asymptomatic *C. sativa* are categorized at this level. No correlation has been established between viral infection and symptom development, whereas persistent infection and wide distribution are the main characteristics of these viral agents. According to these criteria, level D viruses should not be considered as a threat to *C. sativa* production. Viruses belonging to this level are CanCV and CasaMV1. Both are considered persistent viruses that cannot spread from cell to cell unless by cell division, so all host cells are potentially infected, and transmission is essentially vertical. They generally have low titers and no detectable negative impacts on their hosts.

Mitoviruses were previously detected only in fungi, but recently, it became possible to identify them from different public transcriptome datasets of different plants (e.g., hop, hemp, and sugar beet) [50]. For some of these, viral replication has been experimentally demonstrated excluding mitochondrial or nuclear genome endogenization events [51]. This has not yet been conducted for CasaMV1, whose genome has been reconstructed from transcriptomic data.

CanCV was casually discovered by Ziegler et al. (2012) [9] in hemp plants and subsequently detected in almost every plant tested. Righetti et al. (2018) [10] tried to further characterize the virus trying to find a correlation with the HSV, but no significant correlation was found. Cryptic viruses belonging to the *Partitiviridae* family are generally considered not able to cause any symptoms, and some have been associated with mutualistic effects. This suggests positive selection during the domestication of horticultural varieties [16]. The capsid protein of pepper cryptic virus 1, for example, has an intrinsically disordered and hypervariable domain between Deltapartitiviruses that plays no structural role. It is hypothesized that it is involved in an evolutionary mechanism that allows it to maintain persistent infections in different plant hosts by establishing a mutualistic interaction [52]. However, the mutualistic and beneficial virus–host effect is not always guaranteed. This is the case for some fungal Partititivirdae that have a pathogenic effect on their hosts and induce reductions in growth and sporulation [16]. The ability of CanCV to establish a mutualistic effect in infected *C. sativa* plants cannot yet be assumed, but it should at least be considered because of the worldwide distribution of the virus among different cannabis varieties selected through different breeding programs.

## 3. Fully Infectious Viral cDNA Clones and Derived Viral Vectors for Functional Genomic Studies in Cannabis

Viruses are natural vectors for delivering genetic material into cells, and, because of their ability to replicate and produce high quantities of virus-derived mRNA, for decades, they have been profitably used in fundamental virology studies or engineered as biotechnological tools. Several RNA or DNA phytoviruses were transformed into fully infectious cDNA clones and exploited as reverse genetics systems for the study of viral factors and host–pathogen interaction. Infectious cDNA clones can be easily modified and reproposed as vectors for the expression of heterologous proteins or to suppress host gene expression by inducing gene silencing (VIGS), aiming for the selective knock-down expression of endogenous genes in various plants [51,53,54].

No infectious cDNA clones derived from a virus that naturally infects cannabis has been used in this plant to date. However, two viral vectors derived from CLCrV [31] and TRV [32] have been developed to perform VIGS experiments in *C. sativa*.

DNA-A and -B of the bipartite CLCrV were cloned into two binary vectors for *A. tumefaciens*-mediated transformation, in the way that the genes of the two genomic DNAs were flanked by two common regions containing the origin of replication, and a multiple cloning site was inserted upstream AL3 gene to allow the introduction of foreign DNA sequences. Gene portions of 300 to 400 base pairs of phytoene desaturase (PDS) and magnesium chelatase subunit I (ChlI) were selected to track, through chlorophyll or carotenoids foliar bleaching, the occurrence of PTGS in the Finola variety. Specifically, the gene portions of PDS and ChlI were chosen using the method described by Xu et al. (2006) [55] to produce efficient siRNAs and, at the same time, with minimal off-target silencing effects. The VIGS capacity of CLCrV was then verified by analyzing the transcriptional levels of PDS and ChlI, which decreased by 73% and 70%, respectively, in agro-infiltrated cannabis plants. However, carotenoids and chlorophyll a and b levels decreased only between 27 and 40%causing faint green leaves with white and yellow spots and not a remarkable foliar photobleaching phenotype [31].

TRV-based vectors have been widely used for VIGS experiments in different plants in which the virus is infectious [56,57]. TRV is a bipartite RNA virus, which is why two different cDNA agroclones (pTRV1 and pTRV2) are used for VIGS: one expresses RNA1 that encodes the viral functions of replication and movement, while the other harbors RNA2 deprived of the 2b and 2c coding sequence and carrying an MCS downstream of the coat protein sequence. Alter et al. (2022) [32] inserted three different gene portions of PDS into pTRV2 and then performed VIGS examinations in medical cannabis genetic lines for cannabinoid productions. Despite the low ability of infectious TRV clones to induce systemic infection, observed mainly in glandular trichomes, intense and widespread symptoms of leaf photobleaching were observed in the MF-219 gene line, depending on the viral construct chosen, with transcript levels of PDS lower by more than 90% compared to control plants [32].

## 4. Concluding Remarks

**Figure 3:**
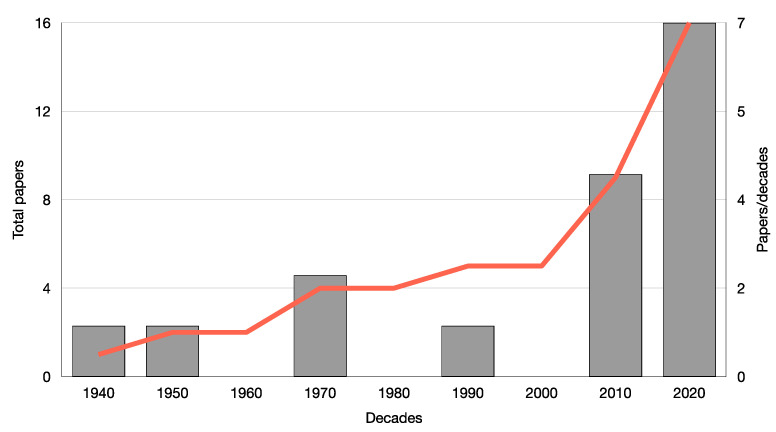
Scientific papers published per decade about virus and viroid infections in *C. sativa* since 1940.

Interest in *C. sativa* is rising in different economic sectors (e.g., medical, industrial, bio-building, cosmetic, food, etc.) [58,59], leading to higher acreage cultivation and to a higher exchange of plant materials for breeding purposes. In addition, plants for high-quality products (e.g., cannabinoids) are often obtained through agamic multiplication from mother plants that can potentially be infected. The combination of these conditions has facilitated the spread of viruses and viroids as evidenced by subsequent increases in published reports of viral infections (Figure 3), refuting the old assumption that cannabis is tolerant or even resistant to viruses [60]. The wide application of modern HTS will undoubtedly help to further characterize the cannabis virome by identifying unreported viruses and viroids that infect this plant. The ability of HTS to generate a large number of sequences and assemble multiple viral genomes from the same biological sample makes this technique an ideal solution for the generic identification of unknown or very different viruses. For the same reason, HTS can be used in viral population studies to analyze inter- or intra-host diversity and dynamics. However, the high investigative power of this technique alone is not sufficient to biologically characterize a pathogenic virus by distinguishing it from a commensal. Therefore, HTS studies must be followed by others aimed at determining the causal relationship between the symptoms described and the occurrence of the infectious agent [61]. This is essential to making effective management and risk assessment decisions. A phytosanitary harmonized diagnostic protocol for the early detection of viruses in *C. sativa* is not currently available, at least in Europe. Private companies can offer screening tests for the detection of the main viruses and viroids reported infecting this plant, but protocols are not shared and there are no standard or validated ones. Therefore, harmonization of protocols is highly needed to develop a reliable shared procedure for the early detection of known viruses, reducing the risk of circulation of infected plant materials. The proposed virus and viroid categorization system is intended to establish a guide for classification among viral infections in cannabis by indicating those that are the most threatening. Our opinion is that future breeding programs for the development of new varieties resistant or tolerant to viruses and viroids belonging to level A should be considered a priority.

From being considered as a possible phytosanitary threat to cannabis, viruses also represent an effective biotechnological tool for studies aimed at the genetic improvement of the plant. Indeed, the use of infectious cDNA clones of cannabis-infecting viruses allows the delivery of genetic material without the need for transformation and thus the creation of genetically modified plants. VIGS experiments have become a standard for studies of functional genetics through a reverse genetics approach. The main advantage of VIGS is that it is an economical and time-saving technique that ensures quick phenotype generation without the need for stable plant transformation. Therefore, even in *C. sativa*, it is possible to use infectious viral cDNA clones to induce the PTGS of target genes by overcoming the difficulty of applying other genomic editing techniques in this plant. Despite recent successes in applying VIGS in cannabis, several problems that may limit its use may remain. Indeed, the viral vector may alter the plant’s metabolism by distorting the resultant phenotype. Moreover, the insertion of exogenous sequences into the VIGS vector may limit the fitness of the recombinant virus, which can result in genomic deletions after a few replicative cycles. Despite these potential limitations, it is expected that the VIGS technique will increase its scope in the cannabis system by exploiting the ability of VIGS-mediated down expression to be inherited by the infected plant progeny through RNA-directed DNA methylation (RdDM) [62]. RdDM experiments are still in their very early days but may represent a future tool for the genetic improvement of cannabis through stable epigenetic modifications.

## Figures and Tables

**Table 1:** Reported viruses and viroid in *C. sativa* organized alphabetically by their taxonomy. Genome organization and references that reported the infections are shown.

Family	Genus	Virus	Genome Organization	Reported by
*Alphaflexiviridae*	*Potexvirus*	Potato virus x (PVX)	Single ssRNA (+) of 5.9–7 kb; monopartite flexuous filament of 470–580 nm length, 13 nm in diameter with helical symmetry.	[6]
*Bromoviridae*	*Alfamovirus*	Alfalfa mosaic virus (AMV)	Three ssRNA (+): 3.2 kb, 2.6 kb and 2.1 kb; tripartite bacilliform particles of 30–57 nm, 28 nm in diameter.	[6]
	*Cucumovirus*	Cucumber mosaic virus (CMV)	Three ssRNA (+): 3.5 kb, 2.7 kb and 2.3 kb; tripartite in icosahedral particles of 26–35 nm.	[6]
	Candidate *Anulavirus*	Grapevine line pattern virus (GLPV)	Three ssRNA (+): 2.8 kb, 2 kb, and 1.5 kb; tripartite in quasi-spherical particles of 25–35 nm.	[7]
	*Ilarvirus*	Tobacco streak virus (TSV)	Three ssRNA (+): 3.5 kb, 2.9 kb and 2.2 kb; tripartite in isometric particles of 26–36 nm.	[7]
*Potyviridae*	*Potyvirus*	Potato virus Y (PVY)	Single ssRNA (+) of 9.7 kb; monopartite in a flexuous filament of 680–900 nm and 13 nm in diameter with helical symmetry. a single positive ssRNA of 9.7 kb.	[6,8]
*Secoviridae*	*Nepovirus*	Arabis mosaic virus (ArMV)	Two ssRNA (+) of 9–13 kb; bipartite in a non-enveloped icosahedral particle of 25–30 nm in diameter.	[6]
		Raspberry ringspot virus (RRSV)		[6]
	*Fabavirus*	Broad bean wilt virus (BBWV)		[6]
*Tospoviridae*	*Orthotospovirus*	Tomato spotted wilt virus (TSWV)	Three ssRNA (−): 8.8 kb (L), 4.8 kb (M), and 2.9 kb (S); monopartite spherical virions of 80–120 nm in diameter embedded in a lipid bilayered envelope.	[6]
*Partitiviridae*	*Betapartitivirus*	Cannabis cryptic virus (CanCV)	Two dsRNA: 2.3 kb and 2.2 kb; bipartite in an icosahedral virion of 30–35 nm in diameter	[9,10]
*Closteroviridae*	*Crinivirus*	Lettuce chlorosis virus (LCV)	Two ssRNA (+): both of 8.6 kb; bipartite in helically constructed filamentous particles of 650–850 nm in diameter.	[11]
*Geminiviridae*	*Curtovirus*	Beet curly top virus (BCTV)	Single ssDNA of 2.9–3.0 kb; “geminated” particles consisting of two twinned incomplete icosahedra with average size of 22 × 38 nm.	[12,13]
*Tombusviridae*	Unclassified *Umbravirus*	Opuntia umbra-like virus (OULV)	Single ssRNA(+) of 2.9 kb	[7]
Unclassified	Unclassified	Citrus yellow vein-associated virus (CYVaV)	Single ssRNA (+) of 2.6 kb	[7]
*Pospiviroidae*	*Cocadviroid*	Hop latent viroid (HLVd)	Circular ssRNA of 256 nt with a stable secondary structure of rod-like or quasi-rod-like conformation.	[14,15]
*Mitoviridae*	*Mitovirus*	Cannabis mitovirus 1 (CasaMV1)	Non-encapsidated ssRNA (+) of 2.8 kb.	[7,16]

**Table 2:** Main characteristic of viruses and viroid infecting *C. sativa* organized by level of threat (from A to D). Information about biology such as transmission and eventual vector alongside seed transmissibility is presented. NCBI taxonomy ID, symptoms described in infected cannabis plants and, and proof of infection are shown.

Virus	NCBI Taxonomy ID (txid)	Transmission/Vector	Seed Transmissible	Symptoms	Proof of Infection	Threat Level
Lettuce chlorosis virus (LCV)	642478	Horizontal—Whiteflies, semi-persistent	No	Complete yellowing and chlorotic lesion	Detected from infected symptomatic field plants	A
Beet curly top virus (BCTV)	10840	Horizontal—Leafhopper, circulative, persistent non propagative	No	Mosaic, mottle, deformation, and wrinkling	Detected from infected symptomatic field plants	A
Hop latent viroid (HLVd)	12907	Horizontal—Mechanical	No	Brittle stems, reduced flower mass, and trichomes. Reduced rooting success rate	Detected from infected symptomatic field plants	A
Potato virus x (PVX)	12183	Horizontal—Mechanical	No	Mosaic on the interveinal part of the leaves	Experimental mechanical inoculation	B
Alfalfa mosaic virus (AMV)	12321	Vertical/Horizontal—Aphids, non-persistent	Yes	Light green mottling, clearing of leaf veins, and wrinkling of newer leaves	Experimental mechanical inoculation	B
Cucumber mosaic virus (CMV)	12305	Vertical/Horizontal—Aphids, non-persistent	Yes	Leaves deformation	Experimental mechanical inoculation	B
Potato virus Y (PVY)	12216	Horizontal—Aphids, non-persistent	No	Light mottling, mosaic, and leaves wrinkling	Experimental mechanical inoculation and vector transmission trials	B
Arabis mosaic virus (ArMV)	12271	Vertical/Horizontal—Nematode and pollen	Yes	Green-yellow mosaic on younger leaves	Experimental mechanical inoculation	B
Raspberry ringspot virus (RRSV)	12809	Vertical/Horizontal—Nematode and pollen	Yes	Green-yellow mosaic and leaf deformation	Experimental mechanical inoculation	B
Broad bean wilt virus (BBWV)	95622	Horizontal—Aphids, non-persistent	No	Green-yellow mosaic on younger leaves	Experimental mechanical inoculation	B
Tomato spotted wilt virus (TSWV)	3052585	Horizontal—Thrips	No	Light green mottling	Experimental mechanical inoculation	B
Grapevine line pattern virus (GLPV)	2741672	Unknown—Aphids, non-persistent	Yes	No correlation	Detected from infected symptomatic field plants	C
Tobacco streak virus (TSV)	12317	Vertical/Horizontal—Thrip	Yes	No correlation	Detected from infected symptomatic plants	C
Opuntia umbra-like virus (OULV)	2283799	Unknown	No information	No correlation	Detected from infected symptomatic field plants	C
Citrus yellow vein-associated virus (CYVaV)	1297894	Unknown	No information	No correlation	Detected from infected symptomatic field plants	C
Cannabis cryptic virus (CanCV)	1115692	Vertical—Unknown	Yes	None observed	Detected from symptomatic and asymptomatic infected plants	D
Cannabis mitovirus 1 (CasaMV1)	2080458	Vertical—Unknown	Yes	None observed	Detected from symptomatic and asymptomatic infected plants	D

## Data Availability

Not applicable.

## Supplementary Materials

The following supporting information can be downloaded at: https://www.mdpi.com/article/10.3390/v15071532/s1, Table S1: NCBI accession number of viruses and viroids that have been detected and sequenced in *Cannabis sativa* L.
