# Supplementary material for: A Guide to Cannabis Virology: From the Virome Investigation to the Development of Viral Biotechnological Tools
Source: Viruses. 2023 Jul 12;15(7):1532. doi: 10.3390/v15071532 (PMC10384868; doi:10.3390/v15071532)
Supplement: Supplementary file 1 [file viruses-15-01532-s001.zip › viruses-2475174-supplementary.pdf]

Accession	Geo_Location	Host	Length	Nuc_Completeness	Organism_Name	Release_Date	Submitters
OQ628293	USA	Cannabis sativa	2932	complete	Beet curly top virus	23/04/23	Schoener,J.L., Wang,S.
OQ068391	USA: WA	Cannabis sativa	2929	complete	Beet curly top virus	28/02/23	Jarugula,S., Wagstaff,C., Mitra,A., Crowder,D., Gang,D., Rayapati,N., Crowder,D.W., Gang,D.R., Rayapati,N.A.
OQ068392	USA: WA	Cannabis sativa	1715	partial	Beet curly top virus	28/02/23	Jarugula,S., Wagstaff,C., Mitra,A., Crowder,D., Gang,D., Rayapati,N., Crowder,D.W., Gang,D.R., Rayapati,N.A.
OL624873	USA: Fresno	Cannabis sativa	2931	complete	Beet curly top virus	02/05/22	Melgarejo,T.A., Chen,L.F., Rojas,M.J., Schilder,A., Gilbertson,R., Gilbertson,R.L.
OL624874	USA: San Bernardino	Cannabis sativa	2930	complete	Beet curly top virus	02/05/22	Melgarejo,T.A., Chen,L.F., Rojas,M.J., Schilder,A., Gilbertson,R., Gilbertson,R.L.
OL624875	USA: San Bernardino	Cannabis sativa	2930	complete	Beet curly top virus	02/05/22	Melgarejo,T.A., Chen,L.F., Rojas,M.J., Schilder,A., Gilbertson,R., Gilbertson,R.L.
MW604759	USA: Mesa, CO	Cannabis sativa	469	partial	Beet curly top virus	16/06/21	Chiginsky,J., Albrecht,T., Nachappa,P.
MW604760	USA: Mesa, CO	Cannabis sativa	470	partial	Beet curly top virus	16/06/21	Chiginsky,J., Albrecht,T., Nachappa,P.
MW604761	USA: Pueblo, CO	Cannabis sativa	458	partial	Beet curly top virus	16/06/21	Chiginsky,J., Albrecht,T., Nachappa,P.
MW604762	USA: Rio Blanco, CO	Cannabis sativa	446	partial	Beet curly top virus	16/06/21	Chiginsky,J., Albrecht,T., Nachappa,P.
MW604763	USA: Delta, CO	Cannabis sativa	458	partial	Beet curly top virus	16/06/21	Chiginsky,J., Albrecht,T., Nachappa,P.
MW604764	USA: Delta, CO	Cannabis sativa	410	partial	Beet curly top virus	16/06/21	Chiginsky,J., Albrecht,T., Nachappa,P.
MW604765	USA: Garfield, CO	Cannabis sativa	400	partial	Beet curly top virus	16/06/21	Chiginsky,J., Albrecht,T., Nachappa,P.
MW604766	USA: Larimer, CO	Cannabis sativa	479	partial	Beet curly top virus	16/06/21	Chiginsky,J., Albrecht,T., Nachappa,P.
MW604767	USA: Conejos, CO	Cannabis sativa	467	partial	Beet curly top virus	16/06/21	Chiginsky,J., Albrecht,T., Nachappa,P.
MW604768	USA: Montezuma, CO	Cannabis sativa	465	partial	Beet curly top virus	16/06/21	Chiginsky,J., Albrecht,T., Nachappa,P.
MW604769	USA: Rio Blanco, CO	Cannabis sativa	477	partial	Beet curly top virus	16/06/21	Chiginsky,J., Albrecht,T., Nachappa,P.
MW604770	USA: Larimer, CO	Cannabis sativa	479	partial	Beet curly top virus	16/06/21	Chiginsky,J., Albrecht,T., Nachappa,P.
MW604771	USA: El Paso, CO	Cannabis sativa	478	partial	Beet curly top virus	16/06/21	Chiginsky,J., Albrecht,T., Nachappa,P.
MW604772	USA: Garfield, CO	Cannabis sativa	459	partial	Beet curly top virus	16/06/21	Chiginsky,J., Albrecht,T., Nachappa,P.
MW604773	USA: Montezuma, CO	Cannabis sativa	401	partial	Beet curly top virus	16/06/21	Chiginsky,J., Albrecht,T., Nachappa,P.
MW604774	USA: Montrose, CO	Cannabis sativa	468	partial	Beet curly top virus	16/06/21	Chiginsky,J., Albrecht,T., Nachappa,P.
MW604775	USA: El Paso, CO	Cannabis sativa	466	partial	Beet curly top virus	16/06/21	Chiginsky,J., Albrecht,T., Nachappa,P.
MW604776	USA: Montrose, CO	Cannabis sativa	480	partial	Beet curly top virus	16/06/21	Chiginsky,J., Albrecht,T., Nachappa,P.
MW604777	USA: Weld, CO	Cannabis sativa	411	partial	Beet curly top virus	16/06/21	Chiginsky,J., Albrecht,T., Nachappa,P.
MW713376	USA: Otero, CO	Cannabis sativa	457	partial	Beet curly top virus	16/06/21	Chiginsky,J., Nachappa,P.
MT878075	USA: Rio Blanco,CO	Cannabis sativa	2732	partial	Beet curly top virus	17/01/21	Albrecht,T., Chiginsky,J., Nachappa,P., Stenglein,M.

MT878076	USA: Delta, CO	Cannabis sativa	1804	partial	Beet curly top virus	17/01/21	Albrecht,T., Chiginsky,J., Nachappa,P., Stenglein,M.
MT878077	USA: Delta, CO	Cannabis sativa	968	partial	Beet curly top virus	17/01/21	Albrecht,T., Chiginsky,J., Nachappa,P., Stenglein,M.
MT878078	USA: Conejos, CO	Cannabis sativa	1294	partial	Beet curly top virus	17/01/21	Albrecht,T., Chiginsky,J., Nachappa,P., Stenglein,M.
MT878079	USA: Conejos, CO	Cannabis sativa	1483	partial	Beet curly top virus	17/01/21	Albrecht,T., Chiginsky,J., Nachappa,P., Stenglein,M.
MW182244	USA	Cannabis sativa	2929	complete	Beet curly top virus	08/12/20	Dickey,L., Masson,R., Hu,J.
MK803280	USA	Cannabis sativa	2931	complete	Beet curly top virus	27/10/19	Giladi,Y., Hadad,L., Luria,N., Cranshaw,W., Dumbrovsky,A.
MK747245	Israel	Cannabis sativa	8672	complete	Lettuce chlorosis virus	10/09/19	Hadad,L., Luria,N., Smith,E., Sela,N., Lachman,O., Dombrovsky,A.
MK747246	Israel	Cannabis sativa	8601	complete	Lettuce chlorosis virus	10/09/19	Hadad,L., Luria,N., Smith,E., Sela,N., Lachman,O., Dombrovsky,A.
OQ420426	Canada: British Columbia	Cannabis sativa	256	partial	Hop latent viroid	28/06/23	Punja,Z.K., Ni,L.
OQ068390	USA: WA	Cannabis sativa	256	complete	Hop latent viroid	28/02/23	Jarugula,S., Wagstaff,C., Mitra,A., Crowder,D., Gang,D., Rayapati,N., Crowder,D.W., Gang,D.R., Rayapati,N.A.
MZ090889	USA: Delta, CO	Cannabis sativa	236	partial	Hop latent viroid	15/01/22	Chiginsky,J., Langemeier,K., MacWilliams,J., Albrecht,T., Cranshaw,W., Fulladolsa,A.C., Kapuscinski,M., Stenglein,M., Nachappa,P.
MZ090890	USA: Boulder, CO	Cannabis sativa	256	partial	Hop latent viroid	15/01/22	Chiginsky,J., Langemeier,K., MacWilliams,J., Albrecht,T., Cranshaw,W., Fulladolsa,A.C., Kapuscinski,M., Stenglein,M., Nachappa,P.
MZ686448	USA	Cannabis sativa	216	partial	Hop latent viroid	21/12/21	Rivedal,H.M., Funke,C., Frost,K., Frost,K.E.
MK791745	USA	Cannabis sativa	237	partial	Hop latent viroid	10/11/19	Bektas,A., Hardwick,K.M., Waterman,K., Kristof,J.
MK791746	USA	Cannabis sativa	239	partial	Hop latent viroid	10/11/19	Bektas,A., Hardwick,K.M., Waterman,K., Kristof,J.
MK791747	USA	Cannabis sativa	213	partial	Hop latent viroid	10/11/19	Bektas,A., Hardwick,K.M., Waterman,K., Kristof,J.
MK791748	USA	Cannabis sativa	205	partial	Hop latent viroid	10/11/19	Bektas,A., Hardwick,K.M., Waterman,K., Kristof,J.
MK791749	USA	Cannabis sativa	211	partial	Hop latent viroid	10/11/19	Bektas,A., Hardwick,K.M., Waterman,K., Kristof,J.
MK791750	USA	Cannabis sativa	212	partial	Hop latent viroid	10/11/19	Bektas,A., Hardwick,K.M., Waterman,K., Kristof,J.
MK791751	USA	Cannabis sativa	256	complete	Hop latent viroid	10/11/19	Bektas,A., Hardwick,K.M., Waterman,K., Kristof,J.
MK795520	USA	Cannabis sativa	285	complete	Hop latent viroid	09/11/19	Bektas,A., Hardwick,K.M., Waterman,K., Kristof,J.
MK795521	USA	Cannabis sativa	205	complete	Hop latent viroid	09/11/19	Bektas,A., Hardwick,K.M., Waterman,K., Kristof,J.
MK795522	USA	Cannabis sativa	277	complete	Hop latent viroid	09/11/19	Bektas,A., Hardwick,K.M., Waterman,K., Kristof,J.
MK795523	USA	Cannabis sativa	277	complete	Hop latent viroid	09/11/19	Bektas,A., Hardwick,K.M., Waterman,K., Kristof,J.
MK795524	USA	Cannabis sativa	208	complete	Hop latent viroid	09/11/19	Bektas,A., Hardwick,K.M., Waterman,K., Kristof,J.
MK795525	USA	Cannabis sativa	210	complete	Hop latent viroid	09/11/19	Bektas,A., Hardwick,K.M., Waterman,K., Kristof,J.

MK795526	USA	Cannabis sativa	319	complete	Hop latent viroid	09/11/19	Bektas,A., Hardwick,K.M., Waterman,K., Kristof,J.
MK795527	USA	Cannabis sativa	256	complete	Hop latent viroid	09/11/19	Bektas,A., Hardwick,K.M., Waterman,K., Kristof,J.
MK795528	USA	Cannabis sativa	229	complete	Hop latent viroid	09/11/19	Bektas,A., Hardwick,K.M., Waterman,K., Kristof,J.
MK795529	USA	Cannabis sativa	236	complete	Hop latent viroid	09/11/19	Bektas,A., Hardwick,K.M., Waterman,K., Kristof,J.
MK795530	USA	Cannabis sativa	224	complete	Hop latent viroid	09/11/19	Bektas,A., Hardwick,K.M., Waterman,K., Kristof,J.
MK795531	USA	Cannabis sativa	224	complete	Hop latent viroid	09/11/19	Bektas,A., Hardwick,K.M., Waterman,K., Kristof,J.
MK795532	USA	Cannabis sativa	216	complete	Hop latent viroid	09/11/19	Bektas,A., Hardwick,K.M., Waterman,K., Kristof,J.
MK795533	USA	Cannabis sativa	201	complete	Hop latent viroid	09/11/19	Bektas,A., Hardwick,K.M., Waterman,K., Kristof,J.
MK795534	USA	Cannabis sativa	281	complete	Hop latent viroid	09/11/19	Bektas,A., Hardwick,K.M., Waterman,K., Kristof,J.
MK795535	USA	Cannabis sativa	258	complete	Hop latent viroid	09/11/19	Bektas,A., Hardwick,K.M., Waterman,K., Kristof,J.
MK795536	USA	Cannabis sativa	230	complete	Hop latent viroid	09/11/19	Bektas,A., Hardwick,K.M., Waterman,K., Kristof,J.
MK795537	USA	Cannabis sativa	265	complete	Hop latent viroid	09/11/19	Bektas,A., Hardwick,K.M., Waterman,K., Kristof,J.
MK795538	USA	Cannabis sativa	270	complete	Hop latent viroid	09/11/19	Bektas,A., Hardwick,K.M., Waterman,K., Kristof,J.
MK795539	USA	Cannabis sativa	292	complete	Hop latent viroid	09/11/19	Bektas,A., Hardwick,K.M., Waterman,K., Kristof,J.
MK795540	USA	Cannabis sativa	292	complete	Hop latent viroid	09/11/19	Bektas,A., Hardwick,K.M., Waterman,K., Kristof,J.
MK795541	USA	Cannabis sativa	292	complete	Hop latent viroid	09/11/19	Bektas,A., Hardwick,K.M., Waterman,K., Kristof,J.
MK795542	USA	Cannabis sativa	209	complete	Hop latent viroid	09/11/19	Bektas,A., Hardwick,K.M., Waterman,K., Kristof,J.
MK795543	USA	Cannabis sativa	213	complete	Hop latent viroid	09/11/19	Bektas,A., Hardwick,K.M., Waterman,K., Kristof,J.
MK795544	USA	Cannabis sativa	209	complete	Hop latent viroid	09/11/19	Bektas,A., Hardwick,K.M., Waterman,K., Kristof,J.
MK795545	USA	Cannabis sativa	213	complete	Hop latent viroid	09/11/19	Bektas,A., Hardwick,K.M., Waterman,K., Kristof,J.
MK795546	USA	Cannabis sativa	217	complete	Hop latent viroid	09/11/19	Bektas,A., Hardwick,K.M., Waterman,K., Kristof,J.
MK795547	USA	Cannabis sativa	220	complete	Hop latent viroid	09/11/19	Bektas,A., Hardwick,K.M., Waterman,K., Kristof,J.
MK795548	USA	Cannabis sativa	258	complete	Hop latent viroid	09/11/19	Bektas,A., Hardwick,K.M., Waterman,K., Kristof,J.
MK795549	USA	Cannabis sativa	251	complete	Hop latent viroid	09/11/19	Bektas,A., Hardwick,K.M., Waterman,K., Kristof,J.
MK795550	USA	Cannabis sativa	246	complete	Hop latent viroid	09/11/19	Bektas,A., Hardwick,K.M., Waterman,K., Kristof,J.
MK795551	USA	Cannabis sativa	284	complete	Hop latent viroid	09/11/19	Bektas,A., Hardwick,K.M., Waterman,K., Kristof,J.
MK795552	USA	Cannabis sativa	218	complete	Hop latent viroid	09/11/19	Bektas,A., Hardwick,K.M., Waterman,K., Kristof,J.
MK795553	USA	Cannabis sativa	270	complete	Hop latent viroid	09/11/19	Bektas,A., Hardwick,K.M., Waterman,K., Kristof,J.

MK795554	USA	Cannabis sativa	233	complete	Hop latent viroid	09/11/19	Bektas,A., Hardwick,K.M., Waterman,K., Kristof,J.
MK795555	USA	Cannabis sativa	238	complete	Hop latent viroid	09/11/19	Bektas,A., Hardwick,K.M., Waterman,K., Kristof,J.
MK795556	USA	Cannabis sativa	238	complete	Hop latent viroid	09/11/19	Bektas,A., Hardwick,K.M., Waterman,K., Kristof,J.
MK795557	USA	Cannabis sativa	233	complete	Hop latent viroid	09/11/19	Bektas,A., Hardwick,K.M., Waterman,K., Kristof,J.
MK795558	USA	Cannabis sativa	228	complete	Hop latent viroid	09/11/19	Bektas,A., Hardwick,K.M., Waterman,K., Kristof,J.
MK795559	USA	Cannabis sativa	246	complete	Hop latent viroid	09/11/19	Bektas,A., Hardwick,K.M., Waterman,K., Kristof,J.
MK795560	USA	Cannabis sativa	232	complete	Hop latent viroid	09/11/19	Bektas,A., Hardwick,K.M., Waterman,K., Kristof,J.
MK795561	USA	Cannabis sativa	257	complete	Hop latent viroid	09/11/19	Bektas,A., Hardwick,K.M., Waterman,K., Kristof,J.
MK795562	USA	Cannabis sativa	214	complete	Hop latent viroid	09/11/19	Bektas,A., Hardwick,K.M., Waterman,K., Kristof,J.
MK795563	USA	Cannabis sativa	287	complete	Hop latent viroid	09/11/19	Bektas,A., Hardwick,K.M., Waterman,K., Kristof,J.
MK795564	USA	Cannabis sativa	233	complete	Hop latent viroid	09/11/19	Bektas,A., Hardwick,K.M., Waterman,K., Kristof,J.
MK795565	USA	Cannabis sativa	225	complete	Hop latent viroid	09/11/19	Bektas,A., Hardwick,K.M., Waterman,K., Kristof,J.
MK795566	USA	Cannabis sativa	240	complete	Hop latent viroid	09/11/19	Bektas,A., Hardwick,K.M., Waterman,K., Kristof,J.
MK795567	USA	Cannabis sativa	246	complete	Hop latent viroid	09/11/19	Bektas,A., Hardwick,K.M., Waterman,K., Kristof,J.
MK795568	USA	Cannabis sativa	212	complete	Hop latent viroid	09/11/19	Bektas,A., Hardwick,K.M., Waterman,K., Kristof,J.
MK795569	USA	Cannabis sativa	242	complete	Hop latent viroid	09/11/19	Bektas,A., Hardwick,K.M., Waterman,K., Kristof,J.
MK795570	USA	Cannabis sativa	204	complete	Hop latent viroid	09/11/19	Bektas,A., Hardwick,K.M., Waterman,K., Kristof,J.
MK795571	USA	Cannabis sativa	257	complete	Hop latent viroid	09/11/19	Bektas,A., Hardwick,K.M., Waterman,K., Kristof,J.
MK795572	USA	Cannabis sativa	294	complete	Hop latent viroid	09/11/19	Bektas,A., Hardwick,K.M., Waterman,K., Kristof,J.
MK795573	USA	Cannabis sativa	241	complete	Hop latent viroid	09/11/19	Bektas,A., Hardwick,K.M., Waterman,K., Kristof,J.
MK795574	USA	Cannabis sativa	233	complete	Hop latent viroid	09/11/19	Bektas,A., Hardwick,K.M., Waterman,K., Kristof,J.
MK795575	USA	Cannabis sativa	205	complete	Hop latent viroid	09/11/19	Bektas,A., Hardwick,K.M., Waterman,K., Kristof,J.
MK795576	USA	Cannabis sativa	252	complete	Hop latent viroid	09/11/19	Bektas,A., Hardwick,K.M., Waterman,K., Kristof,J.
MK795577	USA	Cannabis sativa	256	complete	Hop latent viroid	09/11/19	Bektas,A., Hardwick,K.M., Waterman,K., Kristof,J.
MK795578	USA	Cannabis sativa	220	complete	Hop latent viroid	09/11/19	Bektas,A., Hardwick,K.M., Waterman,K., Kristof,J.
MK795579	USA	Cannabis sativa	222	complete	Hop latent viroid	09/11/19	Bektas,A., Hardwick,K.M., Waterman,K., Kristof,J.
MK795580	USA	Cannabis sativa	275	complete	Hop latent viroid	09/11/19	Bektas,A., Hardwick,K.M., Waterman,K., Kristof,J.
MK795581	USA	Cannabis sativa	257	complete	Hop latent viroid	09/11/19	Bektas,A., Hardwick,K.M., Waterman,K., Kristof,J.

MK795582	USA	Cannabis sativa	214	complete	Hop latent viroid	09/11/19	Bektas,A., Hardwick,K.M., Waterman,K., Kristof,J.
MK795583	USA	Cannabis sativa	255	complete	Hop latent viroid	09/11/19	Bektas,A., Hardwick,K.M., Waterman,K., Kristof,J.
MK795584	USA	Cannabis sativa	228	complete	Hop latent viroid	09/11/19	Bektas,A., Hardwick,K.M., Waterman,K., Kristof,J.
MK795585	USA	Cannabis sativa	243	complete	Hop latent viroid	09/11/19	Bektas,A., Hardwick,K.M., Waterman,K., Kristof,J.
MK795586	USA	Cannabis sativa	273	complete	Hop latent viroid	09/11/19	Bektas,A., Hardwick,K.M., Waterman,K., Kristof,J.
MK795587	USA	Cannabis sativa	243	complete	Hop latent viroid	09/11/19	Bektas,A., Hardwick,K.M., Waterman,K., Kristof,J.
MK795588	USA	Cannabis sativa	210	complete	Hop latent viroid	09/11/19	Bektas,A., Hardwick,K.M., Waterman,K., Kristof,J.
MK795589	USA	Cannabis sativa	254	complete	Hop latent viroid	09/11/19	Bektas,A., Hardwick,K.M., Waterman,K., Kristof,J.
MK795590	USA	Cannabis sativa	230	complete	Hop latent viroid	09/11/19	Bektas,A., Hardwick,K.M., Waterman,K., Kristof,J.
MK795591	USA	Cannabis sativa	258	complete	Hop latent viroid	09/11/19	Bektas,A., Hardwick,K.M., Waterman,K., Kristof,J.
MK795592	USA	Cannabis sativa	244	complete	Hop latent viroid	09/11/19	Bektas,A., Hardwick,K.M., Waterman,K., Kristof,J.
MK795593	USA	Cannabis sativa	254	complete	Hop latent viroid	09/11/19	Bektas,A., Hardwick,K.M., Waterman,K., Kristof,J.
MK795594	USA	Cannabis sativa	268	complete	Hop latent viroid	09/11/19	Bektas,A., Hardwick,K.M., Waterman,K., Kristof,J.
MK795595	USA	Cannabis sativa	268	complete	Hop latent viroid	09/11/19	Bektas,A., Hardwick,K.M., Waterman,K., Kristof,J.
MK795596	USA	Cannabis sativa	268	complete	Hop latent viroid	09/11/19	Bektas,A., Hardwick,K.M., Waterman,K., Kristof,J.
MK795597	USA	Cannabis sativa	252	complete	Hop latent viroid	09/11/19	Bektas,A., Hardwick,K.M., Waterman,K., Kristof,J.
MK795598	USA	Cannabis sativa	217	complete	Hop latent viroid	09/11/19	Bektas,A., Hardwick,K.M., Waterman,K., Kristof,J.
MK795599	USA	Cannabis sativa	232	complete	Hop latent viroid	09/11/19	Bektas,A., Hardwick,K.M., Waterman,K., Kristof,J.
MK795600	USA	Cannabis sativa	216	complete	Hop latent viroid	09/11/19	Bektas,A., Hardwick,K.M., Waterman,K., Kristof,J.
MK795601	USA	Cannabis sativa	216	complete	Hop latent viroid	09/11/19	Bektas,A., Hardwick,K.M., Waterman,K., Kristof,J.
MK795602	USA	Cannabis sativa	220	complete	Hop latent viroid	09/11/19	Bektas,A., Hardwick,K.M., Waterman,K., Kristof,J.
MK795603	USA	Cannabis sativa	245	complete	Hop latent viroid	09/11/19	Bektas,A., Hardwick,K.M., Waterman,K., Kristof,J.
MK795604	USA	Cannabis sativa	227	complete	Hop latent viroid	09/11/19	Bektas,A., Hardwick,K.M., Waterman,K., Kristof,J.
MK795605	USA	Cannabis sativa	252	complete	Hop latent viroid	09/11/19	Bektas,A., Hardwick,K.M., Waterman,K., Kristof,J.
MK795606	USA	Cannabis sativa	243	complete	Hop latent viroid	09/11/19	Bektas,A., Hardwick,K.M., Waterman,K., Kristof,J.
MK795607	USA	Cannabis sativa	212	complete	Hop latent viroid	09/11/19	Bektas,A., Hardwick,K.M., Waterman,K., Kristof,J.
MK795608	USA	Cannabis sativa	264	complete	Hop latent viroid	09/11/19	Bektas,A., Hardwick,K.M., Waterman,K., Kristof,J.
MK795609	USA	Cannabis sativa	253	complete	Hop latent viroid	09/11/19	Bektas,A., Hardwick,K.M., Waterman,K., Kristof,J.

MK795610	USA	Cannabis sativa	246	complete	Hop latent viroid	09/11/19	Bektas,A., Hardwick,K.M., Waterman,K., Kristof,J.
MK795611	USA	Cannabis sativa	227	complete	Hop latent viroid	09/11/19	Bektas,A., Hardwick,K.M., Waterman,K., Kristof,J.
MK795612	USA	Cannabis sativa	269	complete	Hop latent viroid	09/11/19	Bektas,A., Hardwick,K.M., Waterman,K., Kristof,J.
MK795613	USA	Cannabis sativa	229	complete	Hop latent viroid	09/11/19	Bektas,A., Hardwick,K.M., Waterman,K., Kristof,J.
MK795614	USA	Cannabis sativa	247	complete	Hop latent viroid	09/11/19	Bektas,A., Hardwick,K.M., Waterman,K., Kristof,J.
MK795615	USA	Cannabis sativa	259	complete	Hop latent viroid	09/11/19	Bektas,A., Hardwick,K.M., Waterman,K., Kristof,J.
MK795616	USA	Cannabis sativa	251	complete	Hop latent viroid	09/11/19	Bektas,A., Hardwick,K.M., Waterman,K., Kristof,J.
MK795617	USA	Cannabis sativa	263	complete	Hop latent viroid	09/11/19	Bektas,A., Hardwick,K.M., Waterman,K., Kristof,J.
MK795618	USA	Cannabis sativa	248	complete	Hop latent viroid	09/11/19	Bektas,A., Hardwick,K.M., Waterman,K., Kristof,J.
MK795619	USA	Cannabis sativa	263	complete	Hop latent viroid	09/11/19	Bektas,A., Hardwick,K.M., Waterman,K., Kristof,J.
MK795620	USA	Cannabis sativa	266	complete	Hop latent viroid	09/11/19	Bektas,A., Hardwick,K.M., Waterman,K., Kristof,J.
MK795621	USA	Cannabis sativa	219	complete	Hop latent viroid	09/11/19	Bektas,A., Hardwick,K.M., Waterman,K., Kristof,J.
MK795622	USA	Cannabis sativa	237	complete	Hop latent viroid	09/11/19	Bektas,A., Hardwick,K.M., Waterman,K., Kristof,J.
MK795623	USA	Cannabis sativa	237	complete	Hop latent viroid	09/11/19	Bektas,A., Hardwick,K.M., Waterman,K., Kristof,J.
MK795624	USA	Cannabis sativa	231	complete	Hop latent viroid	09/11/19	Bektas,A., Hardwick,K.M., Waterman,K., Kristof,J.
MK795625	USA	Cannabis sativa	221	complete	Hop latent viroid	09/11/19	Bektas,A., Hardwick,K.M., Waterman,K., Kristof,J.
MK795626	USA	Cannabis sativa	260	complete	Hop latent viroid	09/11/19	Bektas,A., Hardwick,K.M., Waterman,K., Kristof,J.
MK795627	USA	Cannabis sativa	239	complete	Hop latent viroid	09/11/19	Bektas,A., Hardwick,K.M., Waterman,K., Kristof,J.
MK795628	USA	Cannabis sativa	251	complete	Hop latent viroid	09/11/19	Bektas,A., Hardwick,K.M., Waterman,K., Kristof,J.
MK795629	USA	Cannabis sativa	284	complete	Hop latent viroid	09/11/19	Bektas,A., Hardwick,K.M., Waterman,K., Kristof,J.
MK795630	USA	Cannabis sativa	251	complete	Hop latent viroid	09/11/19	Bektas,A., Hardwick,K.M., Waterman,K., Kristof,J.
MK795631	USA	Cannabis sativa	276	complete	Hop latent viroid	09/11/19	Bektas,A., Hardwick,K.M., Waterman,K., Kristof,J.
MK795632	USA	Cannabis sativa	243	complete	Hop latent viroid	09/11/19	Bektas,A., Hardwick,K.M., Waterman,K., Kristof,J.
MK795633	USA	Cannabis sativa	250	complete	Hop latent viroid	09/11/19	Bektas,A., Hardwick,K.M., Waterman,K., Kristof,J.
MK795634	USA	Cannabis sativa	245	complete	Hop latent viroid	09/11/19	Bektas,A., Hardwick,K.M., Waterman,K., Kristof,J.
MK795635	USA	Cannabis sativa	285	complete	Hop latent viroid	09/11/19	Bektas,A., Hardwick,K.M., Waterman,K., Kristof,J.
MK795636	USA	Cannabis sativa	231	complete	Hop latent viroid	09/11/19	Bektas,A., Hardwick,K.M., Waterman,K., Kristof,J.
MK795637	USA	Cannabis sativa	276	complete	Hop latent viroid	09/11/19	Bektas,A., Hardwick,K.M., Waterman,K., Kristof,J.

MK795638	USA	Cannabis sativa	226	complete	Hop latent viroid	09/11/19	Bektas,A., Hardwick,K.M., Waterman,K., Kristof,J.
MK795639	USA	Cannabis sativa	247	complete	Hop latent viroid	09/11/19	Bektas,A., Hardwick,K.M., Waterman,K., Kristof,J.
MK795640	USA	Cannabis sativa	222	complete	Hop latent viroid	09/11/19	Bektas,A., Hardwick,K.M., Waterman,K., Kristof,J.
MK795641	USA	Cannabis sativa	228	complete	Hop latent viroid	09/11/19	Bektas,A., Hardwick,K.M., Waterman,K., Kristof,J.
MK795642	USA	Cannabis sativa	206	complete	Hop latent viroid	09/11/19	Bektas,A., Hardwick,K.M., Waterman,K., Kristof,J.
MK795643	USA	Cannabis sativa	226	complete	Hop latent viroid	09/11/19	Bektas,A., Hardwick,K.M., Waterman,K., Kristof,J.
MK795644	USA	Cannabis sativa	226	complete	Hop latent viroid	09/11/19	Bektas,A., Hardwick,K.M., Waterman,K., Kristof,J.
MK774671	USA: California	Cannabis sativa	256	complete	Hop latent viroid	10/06/19	Warren,J.G., Mercado,J., Grace,D.
MH539703	China	Cannabis sativa subsp. sativa	657	partial	Cucumber mosaic virus	16/03/20	Yang,L., Zhao,M.
MW888422	USA: Larimer, CO	Cannabis sativa	2511	partial	Grapevine line pattern virus	15/01/22	Chiginsky,J., Langemeier,K., MacWilliams,J., Albrecht,T., Cranshaw,W., Fulladolsa,A.C., Kapuscinski,M., Stenglein,M., Nachappa,P.
MW888423	USA: Larimer, CO	Cannabis sativa	2374	partial	Grapevine line pattern virus	15/01/22	Chiginsky,J., Langemeier,K., MacWilliams,J., Albrecht,T., Cranshaw,W., Fulladolsa,A.C., Kapuscinski,M., Stenglein,M., Nachappa,P.
MW888424	USA: Larimer, CO	Cannabis sativa	3136	partial	Grapevine line pattern virus	15/01/22	Chiginsky,J., Langemeier,K., MacWilliams,J., Albrecht,T., Cranshaw,W., Fulladolsa,A.C., Kapuscinski,M., Stenglein,M., Nachappa,P.
MT893737	USA: Colorado	Cannabis sativa	2173	partial	Tobacco streak virus	03/03/21	Albrecht,T., Chiginsky,J., Nachappa,P., Stenglein,M.
MT893738	USA: Colorado	Cannabis sativa	2851	partial	Tobacco streak virus	03/03/21	Albrecht,T., Chiginsky,J., Nachappa,P., Stenglein,M.
MT893739	USA: Colorado	Cannabis sativa	3420	partial	Tobacco streak virus	03/03/21	Albrecht,T., Chiginsky,J., Nachappa,P., Stenglein,M.
MT893742	USA: Colorado	Cannabis sativa	455	partial	Cannabis cryptic virus	03/03/21 00:00	Albrecht,T., Chiginsky,J., Nachappa,P., Stenglein,M.
MT893743	USA: Colorado	Cannabis sativa	2322	partial	Cannabis cryptic virus	03/03/21 00:00	Albrecht,T., Chiginsky,J., Nachappa,P., Stenglein,M.
KX709964	Italy	Cannabis sativa	2397	partial	Cannabis cryptic virus	17/09/16 00:00	Menzel,W., Righetti,L., Paris,R., Ratti,C., Onofri,C., Calzolari,D., Knierim,D., Magagnini,G., Pacifico,D., Grassi,G.
KX709965	Italy	Cannabis sativa	2266	partial	Cannabis cryptic virus	17/09/16 00:00	Menzel,W., Righetti,L., Paris,R., Ratti,C., Onofri,C., Calzolari,D., Knierim,D., Magagnini,G., Pacifico,D., Grassi,G.
JN196536	Germany	Cannabis sativa	2420	partial	Cannabis cryptic virus	21/11/11 00:00	Ziegler,A., Matousek,J., Steger,G., Schubert,J.
JN196537	Germany	Cannabis sativa	2290	partial	Cannabis cryptic virus	21/11/11 00:00	Ziegler,A., Matousek,J., Steger,G., Schubert,J.
MT909562	USA: Colorado	Cannabis sativa	2913	partial	Opuntia umbra-like virus	17/01/21 00:00	Albrecht,T., Chiginsky,J., Nachappa,P., Stenglein,M.

MT909563	USA: Colorado	Cannabis sativa	2988	partial	Opuntia umbra-like virus	17/01/21 00:00	Albrecht,T., Chiginsky,J., Nachappa,P., Stenglein,M.
OQ068388	USA: WA	Cannabis sativa	2876	partial	Citrus yellow vein-associated virus	28/02/23 00:00	Jarugula,S., Wagstaff,C., Mitra,A., Crowder,D., Gang,D., Rayapati,N., Crowder,D.W., Gang,D.R., Rayapati,N.A.
OQ068389	USA: WA	Cannabis sativa	1399	partial	Citrus yellow vein-associated virus	28/02/23 00:00	Jarugula,S., Wagstaff,C., Mitra,A., Crowder,D., Gang,D., Rayapati,N., Crowder,D.W., Gang,D.R., Rayapati,N.A.
MT893740	USA: Colorado	Cannabis sativa	2932	partial	Citrus yellow vein-associated virus	03/03/21 00:00	Albrecht,T., Chiginsky,J., Nachappa,P., Stenglein,M.
MT893741	USA: Colorado	Cannabis sativa	2854	partial	Citrus yellow vein-associated virus	03/03/21 00:00	Albrecht,T., Chiginsky,J., Nachappa,P., Stenglein,M.
OQ420425	Canada: British Columbia	Cannabis sativa	2825	partial	Cannabis sativa mitovirus 1	28/06/23 00:00	Punja,Z.K., Ni,L.
MT878080	USA: Rio Blanco, CO	Cannabis sativa	2818	partial	Cannabis sativa mitovirus 1	17/01/21 00:00	Albrecht,T., Chiginsky,J., Nachappa,P., Stenglein,M.
MT878081	USA: Delta, CO	Cannabis sativa	2815	partial	Cannabis sativa mitovirus 1	17/01/21 00:00	Albrecht,T., Chiginsky,J., Nachappa,P., Stenglein,M.
MT878082	USA: Conejos, CO	Cannabis sativa	2819	partial	Cannabis sativa mitovirus 1	17/01/21 00:00	Albrecht,T., Chiginsky,J., Nachappa,P., Stenglein,M.
MT878083	USA: Boulder, CO	Cannabis sativa	2817	partial	Cannabis sativa mitovirus 1	17/01/21 00:00	Albrecht,T., Chiginsky,J., Nachappa,P., Stenglein,M.
MT878084	USA: Boulder, CO	Cannabis sativa	2821	partial	Cannabis sativa mitovirus 1	17/01/21 00:00	Albrecht,T., Chiginsky,J., Nachappa,P., Stenglein,M.
BK010428		Cannabis sativa	2857	partial	Cannabis sativa mitovirus 1	11/07/18 00:00	Nibert,M.L., Vong,M., Fugate,K.K., Debat,H.J.
BK010436		Cannabis sativa	2824	partial	Cannabis sativa mitovirus 1	11/07/18 00:00	Nibert,M.L., Vong,M., Fugate,K.K., Debat,H.J.
BK010437		Cannabis sativa	2825	partial	Cannabis sativa mitovirus 1	11/07/18 00:00	Nibert,M.L., Vong,M., Fugate,K.K., Debat,H.J.
BK010438		Cannabis sativa	2805	partial	Cannabis sativa mitovirus 1	11/07/18 00:00	Nibert,M.L., Vong,M., Fugate,K.K., Debat,H.J.

Table S1: NCBI accession number of viruses and viroids that have been detected and sequenced in *Cannabis sativa* L., general information such as, genome length, completeness of the sequence, date, and the submitters are presented.
